# Extended-Spectrum β-Lactamases in Human Isolates of Multidrug-Resistant Non-typhoidal *Salmonella enterica*

**DOI:** 10.3389/fmicb.2020.592223

**Published:** 2020-12-22

**Authors:** Anahit M. Sedrakyan, Zhanna A. Ktsoyan, Karine A. Arakelova, Magdalina K. Zakharyan, Alvard I. Hovhannisyan, Zaruhi U. Gevorgyan, Armine A. Mnatsakanyan, Elene G. Kakabadze, Khatuna B. Makalatia, Nina A. Chanishvili, Jean-Paul Pirnay, Arsen A. Arakelyan, Rustam I. Aminov

**Affiliations:** ^1^Institute of Molecular Biology, National Academy of Sciences of RA, Yerevan, Armenia; ^2^Department of Clinical Laboratory Diagnostics, Yerevan State Medical University after M. Heratsi, Yerevan, Armenia; ^3^Nork Infectious Clinical Hospital, Ministry of Health of RA, Yerevan, Armenia; ^4^George Eliava Institute of Bacteriophages, Microbiology and Virology, Tbilisi, Georgia; ^5^Department of Exact and Natural Sciences, Ivane Javakhishvili Tbilisi State University, Tbilisi, Georgia; ^6^Queen Astrid Military Hospital, Brussels, Belgium; ^7^The School of Medicine, Medical Sciences and Nutrition, University of Aberdeen, Aberdeen, United Kingdom; ^8^Institute of Fundamental Medicine and Biology, Kazan Federal University, Kazan, Russia

**Keywords:** non-typhoidal *Salmonella*, antimicrobial resistance, extended-spectrum β-lactamases, WGS, antimicrobial resistance genes

## Abstract

A total of 291 non-duplicate isolates of non-typhoidal *Salmonella* (NTS) were collected from the fecal samples of patients with salmonellosis in Armenia and Georgia during 1996–2016. The isolates were tested for resistance to antimicrobials, including extended-spectrum β-lactamases (ESBL). The high prevalence of multidrug-resistance (MDR) and ESBL-producer phenotypes was detected among *Salmonella enterica* subsp. *enterica* serovar Typhimurium (*S*. Typhimurium) isolates collected from patients in Armenia between 1996 and 2016. A total of 36 MDR NTS isolates were subjected to whole genome sequencing (WGS) to determine the genetic background of antimicrobial resistance (AMR) and mobile genetic elements. All ESBL-producing *S*. Typhimurium isolates belonged to the same sequence type (ST328). The ESBL-producer phenotype was associated with plasmid-encoded CTX-M-5 production. A range of other plasmids was associated with resistance to other antimicrobials, including the MDR phenotype.

## Introduction

Non-typhoidal salmonellosis poses a serious public health problem with a significant social and economic impact. According to the WHO estimates, the global burden of foodborne (FB) diarrheal disease agents caused 230,000 deaths in 2010 (WHO, 2015). Among these, non-typhoidal *Salmonella* (NTS) accounted for almost 25% of fatal cases (59,000 deaths, including invasive salmonellosis), with a greater occurrence among children younger than 5 years. Moreover, it was estimated that the number of mortalities due to NTS infections in the world rose to 90,300 cases in 2015 (GBD, 2016). Regional comparisons of the burden of NTS infections highlighted a marked regional variation (WHO, 2015). The Centers for Disease Control and Prevention (CDC) estimates that every year NTS is causing approximately 1.35 million illnesses, 26,500 hospitalizations, and 420 deaths annually in the United States (CDC, 2019). According to the report by the European Food Safety Authority (EFSA) and European Centre for Disease Prevention and Control (ECDPC), the number of confirmed cases of salmonellosis in Europe still remains high, with a total of 91,857 cases reported in 2018 (EFSA and ECDC, 2019). Thus, prevention of *Salmonella* outbreaks as well as efficient therapy of salmonellosis is on the agenda of virtually any healthcare system in the world.

Non-typhoidal *Salmonella* spp. usually causes gastroenteritis characterized by acute onset of fever, diarrhea, abdominal pain, nausea, and sometimes vomiting. Clinical manifestations of salmonellosis depend on a general health, the serotype of infecting *Salmonella enterica*, and the specificity of host-pathogen interaction. The majority of salmonellosis cases are relatively mild or moderate and thus not require specific treatment. However, in some cases, involving children, adults above 65 years and immunocompromised individuals, NTSs can cause severe disease that may be accompanied or followed by complications such as focal infections and bacteremia. In more severe cases, treatment of salmonellosis includes electrolyte replacement, rehydration, and antimicrobial (AM) therapy. Current AM therapy recommendations for enteric infections caused by NTS include third-generation cephalosporins for children and fluoroquinolones, as a therapeutic alternative for infections resistant to third-generation cephems, for adults. However, the emergence and spread of ESBL-producing NTSs as the result of the extensive use of cephalosporins has become a worldwide issue with serious consequences (Philippon et al., 1989; Bradford, 2001; Zhao and Hu, 2013; WHO, 2017; Ur Rahman et al., 2018). Besides, the emergence of non-susceptibility to fluoroquinolones such as ciprofloxacin has been reported among NTSs over the recent decades (Crump et al., 2015; Medalla et al., 2017; CDC, 2018; Song et al., 2018). In the WHO report on the global status of antimicrobial resistance (AMR) and surveillance (WHO, 2014), fluoroquinolone-resistant NTSs were listed among bacteria, drug resistance of which is of international concern. Moreover, control and treatment of salmonellosis is complicated by the dissemination of ESBL-producing strains, which could also acquire resistance to fluoroquinolones (Wong et al., 2014; Crump et al., 2015; Song et al., 2018). Multidrug-resistance (MDR) of NTS strains to the first-line AMs necessitates the use of more toxic or last resort AMs, which is undesirable.

NTS is one of the major FB diseases in Armenia and Georgia. According to the Statistical Committee of the Republic of Armenia, the number of confirmed cases of salmonellosis has been variable over the past decade, with children under 6 years of age comprising at least 50% of patients (SCRA, 2020). A total of 4,392 cases of salmonellosis were reported in Armenia during the period 2010–2019. The incidence of salmonellosis in Armenia has increased from 2016 to 2019, with the incidence rate per 100,000 rising from 12.0 in 2016 to 26.7 in 2019. For the first time in the last decade, a total of 793 cases of salmonellosis were registered in 2019 making the disease the leading foodborne bacterial infection in Armenia. Our previous studies reported the tendency toward the MDR phenotype among the human NTS isolates collected in Armenia between 1996 and 2016 as well as the association of MDR with class 1 integrons (Sedrakyan et al., 2007, 2013a, 2013b, 2017, 2018). According to the National Center for Disease Control and Public Health of Georgia (NCDC), the number of confirmed cases of salmonellosis in this country also varied from year to year (NCDC, 2016). During 2010–2019, the lowest incidence rate per 100,000 was 1.7 (65 cases) in 2010 (NCDC, 2016), while the highest was 8.1 (302 cases) in 2018 (personal communication of Dr. Pikra Shavreshiani from NCDC). A total of 1707 cases of salmonellosis were reported in Georgia during the period 2010-2019 (Vepkhvadze et al., 2015, personal communication of Dr. Pikra Shavreshiani from NCDC). In 2018, among the salmonellosis cases, the outbreaks were associated with *S*. Agona serovars of human origin and *S*. Infantis serovars from food sources. The isolates displayed a high level of AMR. It should be noted here that the occurrence of ESBL-producing NTS isolates of human origin in the neighboring countries Armenia and Georgia has not been studied before. There is also a lack of in-depth characterization of AMR genetics in NTS isolates based on advanced molecular typing approaches, such as the use of whole genome sequencing (WGS). Thus, the main objectives of this work were: (i) identification of known AMR genes via WGS and correlate them with the resistance phenotypes in MDR NTSs recovered from patients in Armenia and Georgia; (ii) in-deep analysis of the prevalence of ESBL-producing phenotypes and genotypes among these isolates.

## Materials and Methods

### Isolation and Identification of NTS Isolates

A total of 291 non-duplicate NTS isolates were obtained from fecal samples of patients with salmonellosis in Armenia and Georgia. In the former, 271 isolates were collected between 1996 and 2016 from patients admitted to the “Nork” Clinical Hospital of Infectious Diseases (Ministry of Health, Yerevan, Armenia). In Georgia, 20 NTS isolates, which were collected between 2014 and 2016, were provided for this study by the Referal Laboratory (RL) at the Republican Clinical Hospital of Infectious Diseases (Georgia). Diagnosis was based on clinical presentations and laboratory analyses. Clinical presentations consistent with gastroenteritis were diarrhea, fever, nausea, vomiting, and abdominal cramps. For this investigation, only patients who were not taking any type of medication including antibiotics in the period from 2 weeks to 1 month before the hospital admission were chosen. In this collection of 291 NTS isolates, a total of 74 isolates were isolated in Armenia in 2016. All available and eligible salmonellosis cases in the hospital in 2016 were involved in the study, including the database of patients (*anamnesis vitae* and *anamnesis morbi*). Sampling in other years, especially from 1996 to 2011, was periodic and thus the database of patients from 1996 to 2011 was available only to a limited extent.

For bacteriological analysis, the fecal samples were processed within 1 h after collection. Presumptive *Salmonella* isolates were identified by standard biochemical tests: fermentation of glucose, negative urease reaction, lysine decarboxylase, negative indole test, H_2_S production, and fermentation of galactitol (dulcitol) (Mikoleit, 2015). Serotypes of isolates were determined in accordance with the White–Kauffmann–Le Minor scheme (Grimont and Weill, 2007), with the use of commercially available polyvalent antisera against flagellar (H) and lipopolysaccharide (O) antigens.

#### Ethics Statement

The study protocol was approved by the Ethics Committee of the Institute of Molecular Biology NAS RA (IORG number 0003427, Assurance number FWA00015042, and IRB number 00004079). Patients were enrolled in the 2016 study after providing written consents. Parental/guardian permissions and consents were obtained when children/minors were enrolled in this study.

### MALDI-TOF MS Identification

To validate the taxonomic position of our isolates by other methods, species level identification of bacterial isolates was performed by matrix assisted laser desorption/ionization – time of flight mass spectrometry (MALDI-TOF MS, MicroFlex^TM^, Bruker Daltonik, MA, United States). Freshly grown bacterial colonies were distributed on a ground steel MALDI target plate using a 1-μl disposable loop. The microbial smears were air-dried and overlaid with 1.0 μL Bruker IVD Matrix HCCA and further air-dried for five min at room temperature. The Bruker MicroFlex instrument was operated using FlexControl 3.0 software (Bruker Daltonik). External calibration (quality control) of the instrument was performed using Bruker’s Bacterial Test Standard (BTS, Bruker Daltonik) (Wattal et al., 2017). Isolates that appeared to be non-*Salmonella* sp. were excluded from further analysis.

### Antimicrobial Susceptibility Determination

Non-typhoidal *Salmonella* isolates were tested for susceptibility toward 14 antimicrobials belonging to 10 different classes. The SOPs were strictly followed, in accordance with the guidelines of the Clinical and Laboratory Standards Institute (CLSI) for standard disk diffusion assays (CLSI, 2017). Muller-Hinton agar (Liofilchem^®^, 2014 s.r.l., Italy) was used. Bacterial inoculum was adjusted to be equivalent to a 0.5 McFarland standard (corresponding to 1–2 × 10^8^ CFU/mL). *Escherichia coli* ATCC 25922 strain was used for quality control. The following AM disks (Liofilchem^®^, 2014 s.r.l., Italy) were used: ampicillin (10 μg), amoxicillin-clavulanic acid (20 μg/10 μg), ceftazidime (30 μg), ceftriaxone (30 μg), chloramphenicol (30 μg), ciprofloxacin (5 μg), gentamicin (10 μg), imipenem (10 μg), nalidixic acid (30 μg), streptomycin (10 μg), sulphonamide (300 μg), tetracycline (30 μg), and trimethoprim-sulfamethoxazole (1.25 μg/23.75 μg). The results of susceptibility testing were interpreted based on the CLSI criteria (CLSI, 2017). Minimum inhibitory concentration (MIC) of azithromycin was determined by agar dilution method according to the CLSI performance standards (CLSI, 2015). Isolates that were resistant to at least three different classes of antimicrobials were considered as MDR.

### Determination of ESBL-Producing Phenotype

The ESBL phenotype was identified by the double-disk test using ceftriaxone and ceftazidime with and without clavulanic acid, according to the guidelines of the CLSI (CLSI, 2017). The results were verified using E-test strips (ESBL CT/CTL; BIOMÉRIEUX) according to the manufacturer’s instructions. Muller-Hinton agar (Liofilchem^®^, 2014 s.r.l., Italy) was used. Inoculations of bacteria were made to be equivalent to a 0.5 McFarland standard. *E. coli* strains ATCC 25922 and ATCC 35218 were used for quality control. *E*-test results were interpreted according to the manufacturer’s instructions (Liofilchem^®^, 2014).

### Bacterial DNA Extraction

Total bacterial DNA was extracted using the MO BIO Laboratories Inc. UltraClean^®^ Microbial DNA Isolation Kit according to the manufacturer’s recommendations. DNA concentration and purity were determined using a Micro Spectrophotometer (model Nano-200, Allsheng). DNA quality was also evaluated by 0.75% agarose (VWR Life Science AMRESCO Agarose RA^TM^) gel electrophoresis. DNA samples were stored in 10 mM Tris (with no EDTA) at −20°C.

### PCR Detection of β-Lactamase (*bla*) Genes

Genes encoding β-lactamases (CTX-M, OXA-1, TEM, CMY2, and SHV) were detected by PCR as described previously (Arlet et al., 1997; Frederic et al., 2002; Olesen et al., 2004; Hasman et al., 2006; Hendriksen et al., 2009; Sugumar et al., 2014). The primers used are listed in Supplementary Table 1.

### Whole Genome Sequencing (WGS) of *Salmonella* Isolates

Genome sequencing was provided by MicrobesNG^1^, which is supported by the BBSRC (grant number BB/L024209/1). Genomic DNA libraries were sequenced using Illumina MiSeq and HiSeq 2500 platforms. Bacterial strains were sequenced using 2 bp × 250 bp paired-end reads at 30× coverage or higher. Reads were adapter-trimmed using Trimmomatic 0.30 with a sliding window quality cut off of Q15 (Bolger et al., 2014). *De novo* assembly of reads was performed using SPAdes version 3.7 (Bankevich et al., 2012), and the contigs were annotated using Prokka 1.11 (Seemann, 2014). Assembly metrics were calculated using QUAST (Gurevich et al., 2013). Taxonomic rank assignment was carried out using the Kraken software (Wood and Salzberg, 2014). Whole genome sequences of MDR isolates of NTS are available in the European Nucleotide Archive (ENA) database under Project PRJEB36290 for isolates from Armenia and Projects PRJNA656406, PRJNA656411, and PRJNA656415 for isolates from Georgia. Accession numbers for individual isolates are listed in Supplementary Table 2.

### Bioinformatics Analyses

Serotypes of NTS strains were identified using a Web-based tool SeqSero^2^ (Zhang et al., 2015). Assignment of isolates to sequence types (ST) was performed using a MLST 2.0 tool (Multi-Locus Sequence Typing), which is publicly available at https://cge.cbs.dtu.dk/services/MLST/ (Larsen et al., 2012). Resistance gene identifier (RGI) tool in the comprehensive antibiotic resistance database CARD^3^ (Alcock et al., 2020) was employed for *in silico* prediction of antibiotic resistance genes. PlasmidFinder 2.1 tool^4^ (Carattoli et al., 2014) was used to detect plasmids. MobileElementFinder (v1.02) tool^5^ (Johansson et al., 2020) was employed for identification of mobile genetic elements and their relation to antimicrobial resistance genes. GIPSy (Soares et al., 2016) tool and IslandViewer4^6^ (Bertelli et al., 2017) webserver were used for prediction of genomic islands. *Salmonella* genomic island 1 (SGI1) and phage-type DT104 were detected based on WGS data using primer sets (Pritchett et al., 2000; Siebor and Neuwirth, 2013, 2014; Yukawa et al., 2015) listed in Supplementary Table 1. Contigs were analyzed using BLAST^7^.

### Statistical Analyses

*P*-value (two-tailed) from Fisher’s exact test was calculated using the on-line GraphPad QuickCalcs resource^8^ (Accessed August 5, 2020) to evaluate statistical differences between the compared groups. *P*-values ≤ 0.05 were considered to be significant.

## Results

### Identification of MDR in NTS Isolates

Antimicrobial susceptibility profile of 291 clinical NTS isolates was determined, with 57 (19.59%) of them displaying the MDR phenotype (Supplementary Table 2). Of these, 54 MDR isolates were from Armenia (19.93%, 54/271) collected in the 1996–2016 surveillance, and three isolates – from Georgia (15%, 3/20), collected in 2016.

A total of 53 MDR isolates were also identified to the species level using MALDI-TOF MS and confirmed as *Salmonella* sp. with score values in range of 2.063–2.48 (Supplementary Table 2). The other four MDR isolates were confirmed as belonging to *S. enterica* based on analysis of WGS data (Supplementary Table 2).

Serotype analyses indicated that *S*. Typhimurium was the most prevalent serotype among the MDR isolates in this study (84.21%, 48/57), followed by *S*. Enteritidis (8.77%, 5/57), *S*. Derby (3.51%, 2/57), *S*. Kentucky (1.75%, 1/57), and *S*. Newport (1.75%, 1/57).

### Antimicrobial Susceptibility of MDR Isolates

Summary of antimicrobial susceptibility profiles among the MDR NTS isolates is presented in Table 1. The results indicate that there is not a single antimicrobial in the panel, to which all MDR NTS isolates are susceptible. NTS MDR isolates are highly susceptible to imipenem (98.25%) and gentamicin (98.25%), followed by trimethoprim-sulfamethoxazole (82.46%), chloramphenicol (78.95%), tetracycline (77.19%), and streptomycin (75.44%). The high level of intermediate resistance among MDR NTS isolates was detected toward amoxicillin-clavulanic acid (52.63%), ceftazidime (47.37%), and ciprofloxacin (38.60%). The highest resistance levels were against nalidixic acid (89.47%), ampicillin (87.72%), and ceftriaxone (71.93%). As for azithromycin, 5.26% (3/57) of the MDR isolates had MIC of 32 μg/mL and 8.77% (5/57) had MIC of ≥64 μg/mL (Supplementary Table 2). The AMR profiles of the MDR NTS isolates were analyzed to reveal the most prevalent drug-resistance patterns (Supplementary Table 2). The most frequent AMR pattern, which included resistance to ampicillin, ceftriaxone and nalidixic acid, was encountered in 71.93% of the isolates (41/57). Of these, 73.17% of isolates (30/41) had an additional resistance to amoxicillin-clavulanic acid and ceftazidime. The most prevalent AMR profile was detected in 17.54% of isolates (10/57, all *S*. Typhimurium) that were resistant to ampicillin, ceftriaxone, and nalidixic acid and showed intermediate resistance to amoxicillin-clavulanic acid and ceftazidime. The second most prevalent drug-resistance profile, which possessed an additional intermediate resistance to ciprofloxacin, was identified in 8.77% of isolates (5/57, also all *S*. Typhimurium). Resistance to five or more classes of AMs was identified in 19 MDR NTS isolates (33.3%, 19/57), consisting of 18 *S*. Typhimurium and one *S*. Newport.

**TABLE 1:** Susceptibility to antimicrobials in multidrug-resistant isolates of non-typhoidal *Salmonella* recovered from patients in Armenia (54 isolates) and Georgia (3 isolates).

Antimicrobial agent	AM class/subclass (CLSI)	Total MDR isolates (*N* = 57), % (N)			*S*. Typhimurium isolates (*N* = 48), % (N)			Isolates with other serotypes (*N* = 9), % (N)			ESBL-producer isolates (*N* = 37), % (N)			ESBL-negative isolates (*N* = 20), % (N)		
						
		S	I	R	S	I	R	S	I	R	S	I	R	S	I	R
Ampicillin (AMP)	Penicillins/Aminopenicillin	8.77 (5)	3.51 (2)	87.72 (50)	6.25 (3)	0.00 (0)	93.75 (45)	22.22 (2)	22.22 (2)	55.56 (5)	0.00 (0)	0.00 (0)	100 (37)	25.00 (5) ↑^b^	10.00 (2)	65.00 (13)
Amoxicillin- Clavulanic acid (AUG)	β-Lactam/ β-lactamase inhibitor combinations	28.07 (16)	52.63 (30)	19.30 (11)	18.75 (9)	58.33 (28)	22.92 (11)	77.78 (7) ↑^a^	22.22 (2)	0.00 (0)	13.51 (5)	70.27 (26)	16.22 (6)	55.00 (11) ↑^b^	20.00 (4)	25.00 (5)
Ceftriaxone (CRO)	Cephems/Cephalosporin III	26.32 (15)	1.75 (1)	71.93 (41)	16.67 (8)	0.00 (0)	83.33 (40)	77.78 (7) ↑^a^	11.11 (1)	11.11 (1)	0.00 (0)	0.00 (0)	100 (37)	75.00 (15) ↑^b^	5.00 (1)	20.00 (4)
Ceftazidime (TZ)	Cephems/Cephalosporin III	38.60 (22)	47.37 (27)	14.04 (8)	29.17 (14)	56.25 (27)	14.58 (7)	88.89 (8) ↑^a^	0.00 (0)	11.11 (1)	10.81 (4)	70.27 (26)	18.92 (7)	90.00 (18) ↑^b^	5.00 (1)	5.00 (1)
Imipenem (IMI)	Penems/Carbapenem	98.25 (56)	1.75 (1)	0.00 (0)	97.92 (47)	2.08 (1)	0.00 (0)	100 (9)	0.00 (0)	0.00 (0)	100 (37)	0.00 (0)	0.00 (0)	95.00 (19)	5.00 (1)	0.00 (0)
Ciprofloxacin (CIP)	Fluoroquinolones	56.14 (32)	38.60 (22)	5.26 (3)	58.33 (28)	41.67 (20)	0.00 (0)	44.44 (4)	22.22 (2)	33.33 (3)	56.76 (21)	43.24 (16)	0.00 (0)	55.00 (11)	30.00 (6)	15.00 (3)
Nalidixic acid (NAL)	Quinolone	10.53 (6)	0.00 (0)	89.47 (51)	8.33 (4)	0.00 (0)	91.67 (44)	22.22 (2)	0.00 (0)	77.78 (7)	0.00 (0)	0.00 (0)	100 (37)	30.00 (6) ↑^b^	0.00 (0)	70.00 (14)
Gentamicin (G)	Aminoglycosides	98.25 (56)	0.00 (0)	1.75 (1)	97.92 (47)	0.00 (0)	2.08 (1)	100 (9)	0.00 (0)	0.00 (0)	97.30 (36)	0.00 (0)	2.70 (1)	100 (20)	0.00 (0)	0.00 (0)
Streptomycin (SM)	Aminoglycosides	75.44 (43)	10.53 (6)	14.04 (8)	79.17 (38)	8.33 (4)	12.50 (6)	55.56 (5)	22.22 (2)	22.22 (2)	89.19 (33)	5.40 (2)	5.40 (2)	50.00 (10) ↓^c^	20.00 (4)	30.00 (6)
Sulfonamide (S3)	Folate pathway inhibitors	63.16 (36)	0.00 (0)	36.84 (21)	66.67 (32)	0.00 (0)	33.33 (16)	44.44 (4)	0.00 (0)	55.56 (5)	78.38 (29)	0.00 (0)	21.62 (8)	35.00 (7) ↓^c^	0.00 (0)	65.00 (13)
Trimethoprim- Sulfamethoxazole (T/S)	Folate pathway inhibitors	82.46 (47)	3.51 (2)	14.04 (8)	83.33 (40)	4.17 (2)	12.50 (6)	77.78 (7)	0.00 (0)	22.22 (2)	86.49 (32)	5.40 (2)	8.11 (3)	75.00 (15)	0.00 (0)	25.00 (5)
Chloramphenicol (C)	Phenicols	78.95 (45)	0.00 (0)	21.05 (12)	79.17 (38)	0.00 (0)	20.83 (10)	77.78 (7)	0.00 (0)	22.22 (2)	89.19 (33)	0.00 (0)	10.81 (4)	60.00 (12) ↓^c^	0.00 (0)	40.00 (8)
Tetracycline (TE)	Tetracyclines	77.19 (44)	0.00 (0)	22.81 (13)	81.25 (39)	0.00 (0)	18.75 (9)	55.56 (5)	0.00 (0)	44.44 (4)	89.19 (33)	0.00 (0)	10.81 (4)	55.00 (11) ↓^c^	0.00 (0)	45.00 (9)

*AM, antimicrobial; CLSI, Clinical and Laboratory Standards Institute; MDR, multidrug-resistant; ESBL, extended-spectrum beta-lactamase; interpretive categories: S, susceptible; I, intermediate; R, resistant. ^*a*^Statistically significant differences between S. Typhimurium isolates and isolates belonging to other serotypes of non-typhoidal Salmonella, two-tailed Fisher’s exact test P-value < 0.005. ^*b*^Statistically significant differences between ESBL-positive and ESBL-negative isolates, two-tailed Fisher’s exact test P-value < 0.005. ^*c*^Statistically significant differences between ESBL-positive and ESBL-negative isolates, two-tailed Fisher’s exact test P-value < 0.05.*

It should be noted here that the most prevalent serotype among the MDR NTS isolates was *S*. Typhimurium (48 out of 57). The remaining MDR isolates of other serotypes (15.79%, 9/57) were still susceptible to imipenem and gentamicin (Table 1). Besides, among the other than *S*. Typhimurium isolates, there was a higher level of susceptibility to ceftazidime (88.89%), amoxicillin-clavulanic acid (77.78%) and ceftriaxone (77.78%). In *S*. Typhimurium the corresponding susceptibility levels were significantly lower (29.17, 18.75, and 16.67%; respectively; *p* < 0.005). On the contrary, we did not find *S*. Typhimurium isolates resistant to ciprofloxacin. This resistance was encountered only in two *S*. Derby and one *S*. Enteritidis isolates. However, the intermediate resistance to ciprofloxacin was identified in 41.67% of *S*. Typhimurium isolates compared to 22.22% among other MDR serotypes. It should be emphasized, that among the 19 isolates displaying resistance to five or more classes of AMs, 18 isolates were *S*. Typhimurium (94.74%, 18/19). Moreover, five *S*. Typhimurium isolates (A_3040, A_3246, A_3725, A_3889, and A_5962) exhibited resistance to eight classes of AMs (Supplementary Table 2). The results indicated that certain serotypes are more prone to acquision of AMR compared to others.

### ESBL-Producing and ESBL-Negative Isolates Detected Phenotypically

All 57 MDR NTS strains were further explored for the resistance to ESBLs. Among these, five were sensitive to all β-lactams and were excluded from further analysis (Supplementary Table 2). From the remaining 52 MDR isolates, 42 displayed resistance to ceftriaxone and the other 10 - to other β-lactams (Supplementary Table 2). We detected a total of 37 NTS isolates displaying ESBL-producing phenotype (64.91%, 37/57). All of them originated from patients in Armenia (68.52%, 37/54), in samples collected between 1996 and 2014. All MDR NTS isolates from Georgia were ESBL-negative (0/3). It should be noted, that all ESBL-producing isolates were identified as *S*. Typhimurium. Thus, the majority of *S*. Typhimurium isolates (37/48, 77.08%) were ESBL-producers. This phenotype was also identified in four out of five *S*. Typhimurium isolates displaying resistance to eight classes of AMs (strains A_3040, A_3246, A_3725, and A_5962). These results indicated the high prevalence of MDR and ESBL-producing phenotypes in clinical isolates of *S*. Typhimurium in Armenia.

All ESBL-producing isolates were not susceptible to ampicillin, ceftriaxone, and nalidixic acid (Table 1). They also displayed resistance toward ceftazidime (10.81%) and amoxicillin-clavulanic acid (13.51%), with a high level of intermediate resistance to these AMs (70.27%). No ESBL-producing isolates were resistant to ciprofloxacin, but 43.24% of them displayed the intermediate resistance to this antibiotic. Thus, the high level of resistance toward the first-line AMs for empiric salmonellosis therapy such as third generation cephalosporins and fluoroquinolones was observed among the ESBL-producers. Alternative treatment options include azithromycin and trimethoprim-sulfamethoxazole. ESBL-producing isolates, however, demonstrated MIC values to azithromycin at ≥32 μg/mL in 16.22% of them (6/37; Supplementary Table 2), while resistance to trimethoprim-sulfamethoxazole was detected in 13.51% (5/37) of them (Table 1). The highest level of susceptibility to other non-β-lactam AMs among ESBL-producers was to gentamicin (97.3%), chloramphenicol (89.19%), streptomycin (89.19%), and tetracycline (89.19%). Of note, ESBL-producing isolates demonstrated a 100% susceptibility toward imipenem. This AM is one of the last-resort therapy option for complicated *Salmonella* infections.

Among the 57 MDR NTS isolates, 20 (35.09%) displayed ESBL-negative phenotype (Supplementary Table 2). These included the following serotypes: *S.* Typhimurium (55%, 11/20), *S.* Enteritidis (25%, 5/20), *S.* Derby (10%, 2/20), *S.* Kentucky (5%, 1/20), and *S.* Newport (5%, 1/20). These isolates were all susceptible to gentamicin (100%, 20/20) and, almost entirely, to imipenem (95%, 19/20) (Table 1). A higher level of susceptibility to β-lactams and quinolones was detected in ESBL-negative isolates compared to ESBL-producers (*p* < 0.005; Table 1). In particular, 90% of these isolates were susceptible to ceftazidime (vs. 10.81% in ESBL-producers), 75% - to ceftriaxone (vs. 0% in ESBL-producers), 55% – to amoxicillin-clavulanic acid (vs. 13.51% in ESBL-producers), 25% - to ampicillin (vs. 0% in ESBL-producers), and 30% - to nalidixic acid (vs. 0% in ESBL-producers). On the contrary, a lower level of susceptibility in ESBL-negative isolates was found toward chloramphenicol (60%), tetracycline (55%), streptomycin (50%), and sulphonamide (35%) compared to ESBL-producers (89.19, 89.19, 89.19, and 78.38%, respectively; *p* < 0.05). The level of susceptibility to ciprofloxacin was similar in these two groups, but three ESBL-negative isolates (15%, 3/20) were resistant to ciprofloxacin (vs. 0% in ESBL-producers). Of note, the ESBL-negative phenotype was identified in one of *S*. Typhimurium isolates (A_3889), which displayed resistance to 8 classes of AMs (Supplementary Table 2). The identical MDR profile was revealed in an ESBL-positive *S*. Typhimurium A_5962 strain.

### PCR Detection of β-Lactamases in MDR NTSs

All 57 MDR NTS isolates in this study were tested by PCR for the presence of β-lactamases (CTX-M, OXA-1, TEM, CMY2, and SHV) (Supplementary Tables 1, 2). The most prevalent β-lactamase was CTX-M detected in 71.93% of MDR NTS isolates (41/57), followed by OXA-1 (12.28%, 7/57), and TEM (3.51%, 2/57). Notably, neither CMY2 nor SHV β-lactamases were detectable in these MDR NTS isolates.

All ESBL-producers displayed the presence of CTX-M (37/37), while it was detectable only in 20% of ESBL-negative isolates (4/20, *p* < 0.0001). On the contrary, there was no significant difference in prevalence of OXA-1, which was detected in 10.81% (4/37) of ESBL-producers and in 15% (3/20) of ESBL-negative isolates. Notably, no TEM β-lactamases were detected in ESBL-producers, while still detectable in 15% (3/20) of ESBL-negative isolates.

### Whole Genome Sequencing of MDR Isolates and *in silico* Multilocus Sequence Typing (MLST)

All MDR isolates from Armenia exhibiting resistance to more than 5 classes of AMs were selected for WGS. Besides, WGS was performed with strains having the most common MDR phenotypes but isolated during different years. All three MDR isolates from Georgia were also subjected to WGS. A total of 36 MDR NTS isolates were subjected to WGS (Supplementary Table 2): 19 ESBL-producing *S*. Typhimurium isolates and 17 ESBL-negative isolates (eight *S*. Typhimurium, five *S*. Enteritidis, two *S*. Derby, one *S*. Kentucky, and one *S*. Newport isolates). Genome sequences are available in the ENA database under the project accession numbers PRJEB36290, PRJNA656406, PRJNA656411, and PRJNA656415. Individual accession numbers are listed in Supplementary Table 2.

The sequence type (ST) of the isolates was determined *in silico* using an MLST 2.0 tool (Larsen et al., 2012). From the 19 ESBL-producers, 18 of them were assigned to ST328, while the remaining ESBL-producer (A_126 isolate that was identified as *S*. Typhimurium by agglutination tests) could not be decidedly typed. The eight ESBL-negative *S*. Typhimurium isolates were classified into three ST types: four isolates to ST328, three – to ST19, and one – to ST36. The ST of the remaining nine ESBL-negative isolates was classified as follows: five *S*. Enteritidis isolates as belonging to ST11, two *S*. Derby isolates – to ST40, one *S*. Kentucky isolate – to ST198, and one *S*. Newport isolate – to ST31. Thus, almost all ESBL-producers (18 out of 19) belonged to the same ST328, whereas ESBL-negative isolates displayed a relatively high degree of genetic variability.

### *In silico* Identification of AMR Genes

Resistance gene identifier (RGI) in CARD (Alcock et al., 2020) was employed for the *in silico* prediction of AMR genes in the genome sequences. Correlation between the AMR genotypes and phenotypes among the MDR isolates is given in Supplementary Table 2. The AMR genes identified and their prevalence in the clinical isolates are summarized in Supplementary Table 3.

### β-Lactam Resistance

As determined by bioinformatics analysis of WGS data, all 19 ESBL-positive isolates possessed the common β-lactam resistance mechanisms. According to CARD, these included the *bla*_CTX–M–__5_ gene and two amino acid substitutions, S357N and D350N, in penicillin binding protein 3 (PBP3), which is encoded by the *ftsI* gene (Misawa et al., 2018) (Table 2). In four isolates (4/19, 21.05%), in addition to the common β-lactam resistance mechanisms, the *bla*_OXA–__1_ gene was identified. In two other isolates, the *bla*_CTX–M–__15_ gene was found as an addition to the common β-lactam resistance mechanisms. And one isolate demonstrated the presence of *bla*_CTX–M–__3_ in addition to the common genetic background determining resistance to β-lactams. All the ESBL-positive isolates sequenced displayed resistance toward ampicillin and ceftriaxone. The majority of these isolates showed intermediate or full resistance against ceftazidime (94.74%, 18/19) and amoxicillin-clavulanic acid (84.21%, 16/19). Notably, the *bla*_OXA–__1_ gene was found in four out of five sequenced ESBL-producing isolates that were resistant to amoxicillin-clavulanic acid.

**TABLE 2:** Profiles of genes conferring resistance to β-lactam antibiotics identified *in silico* in 19 ESBL-producing *S*. Typhimurium isolates from Armenia.

Profile no.	Number of isolates (%)	Isolate ID	Year of isolation	MLST	Antimicrobial resistance phenotype	Antimicrobial resistance genes identified by WGS		
						Resistance to β-lactams	Resistance to quinolones/fluoroquinolones	Resistance to other classes of antimicrobials
1	12 (63.19%)	A_60	1996	328	AMP, CRO, TZ*, NAL	*bla*_CTX–M–__5_, *ftsI* (S357N, D350N)	*gyrA (*D87N*)*	*aac(6*′*)-Iaa*
		A_69	2006	328	AMP, CRO, TZ*, NAL	*bla*_CTX–M–__5_, *ftsI* (S357N, D350N)	*gyrA (*D87N*)*	*aac(6*′*)-Iaa*
		A_105	2011	328	AMP, AUG*, CRO, TZ*, NAL	*bla*_CTX–M–__5_, *ftsI* (S357N, D350N)	*gyrA (*D87N*)*	NI**
		A_115	2011	328	AMP, AUG*, CRO, TZ, CIP*, NAL	*bla*_CTX–M–__5_, *ftsI* (S357N, D350N)	*gyrA (*D87N*)*	*aac(6*′*)-Iaa*
		A_645	2006	328	AMP, AUG*, CRO, TZ*, NAL	*bla*_CTX–M–__5_, *ftsI* (S357N, D350N)	*gyrA (*D87N*)*	*aac(6*′*)-Iaa*
		A_678	2011	328	AMP, AUG*, CRO, TZ*, NAL	*bla*_CTX–M–__5_, *ftsI* (S357N, D350N)	*gyrA (*D87N*)*	*aac(6*′*)-Iaa*
		A_684	1996	328	AMP, CRO, TZ*, NAL	*bla*_CTX–M–__5_, *ftsI* (S357N, D350N)	*gyrA (*D87N*)*	NI
		A_1328	2012	328	AMP, AUG, CRO, TZ*, CIP*, NAL	*bla*_CTX–M–__5_, *ftsI* (S357N, D350N)	*gyrA (*D87N*)*	*aac(6*′*)-Iaa*
		A_3017	2013	328	AMP, AUG*, CRO, TZ*, CIP*, NAL	*bla*_CTX–M–__5_, *ftsI* (S357N, D350N)	*gyrA (*D87N*)*	*aac(6*′*)-Iaa*
		A_3175	2014	328	AMP, AUG*, AZI, CRO, TZ*, CIP*, NAL	*bla*_CTX–M–__5_, *ftsI* (S357N, D350N)	*gyrA (*D87N*)*	*aac(6*′*)-Iaa*
		A_3194	2012	328	AMP, AUG*, AZI, CRO, TZ, CIP*, NAL	*bla*_CTX–M–__5_, *ftsI* (S357N, D350N)	*gyrA (*D87N*)*	*aac(6*′*)-Iaa*
		A_3406	2012	328	AMP, AUG*, AZI, CRO, TZ, CIP*, NAL	*bla*_CTX–M–__5_, *ftsI* (S357N, D350N)	*gyrA (*D87N*)*	*aac(6*′*)-Iaa*
2	4 (21.05%)	A_3040	2014	328	AMP, AUG, CRO, TZ, NAL, C, SM, TE, S3, T/S	*bla*_CTX–M–__5_, *bla*_OXA–__1_, *ftsI* (S357N, D350N)	*gyrA (*D87N*)*	*aac(6*′*)-Iaa, aadA, aph(3*′*)-Ia, cat1, dfrA19, sul1, tet(B), tetR*
		A_3246	2014	328	AMP, AUG, CRO, TZ, CIP*, NAL, C, GEN, SM, S3, T/S, TE	*bla*_CTX–M–__5_, *bla*_OXA–__1_, *ftsI* (S357N, D350N)	*gyrA (*D87N*)*	*aac(6*′*)-Iaa, aadA, ant(2*′′*)-Ia, aph(3*′*)-Ia, catI, catB3, dfrA19, sul1, tet(B), tetR*
		A_3725	2014	328	AMP, AUG, CRO, TZ*, NAL, C, SM*, S3, TE	*bla*_CTX–M–__5_, *bla*_OXA–__1_, *ftsI* (S357N, D350N)	*gyrA (*D87N*)*	*aac(6*′*)-Iaa, aadA, aph(3*′*)-Ia, catI, sul1, tet(B), tetR*
		A_5962	2012	328	AMP, AUG, CRO, TZ*, CIP*, NAL, C, SM*, S3, TE	*bla*_CTX–M–__5_, *bla*_OXA–__1_, *ftsI* (S357N, D350N)	*gyrA (*D87N*)*	*aac(6*′*)-Iaa, aadA, aph(3*′*)-Ia, catI, sul1, tet(B), tetR*
3	2 (10.53%)	A_6004	2013	328	AMP, AUG*, CRO, TZ, CIP*, NAL, S3, T/S*	*bla*_CTX–M–__5_, *bla*_CTX–M–__15_, *ftsI* (S357N, D350N)	*gyrA (*D87N*)*	*aac(6*′*)-Iaa, dfrA5*
		A_8011	2014	328	AMP, AUG*, AZI, CRO, TZ, CIP*, NAL, S3, T/S*	*bla*_CTX–M–__5_, *bla*_CTX–M–__15_, *ftsI* (S357N, D350N)	*gyrA (*D87N*)*	*aac(6*′*)-Iaa, dfrA5*
4	1 (5.26%)	A_126	2011	NI	AMP, AUG*, CRO, NAL, S3, T/S	*bla*_CTX–M–__5_, *bla*_CTX–M–__3_, *ftsI* (S357N, D350N)	*gyrA (*D87N*)*	*aac(6*′*)-Iaa, dfrA14*

ESBL, extended-spectrum beta-lactamase; MDR, multidrug-resistant; NTS, non-typhoidal Salmonella; MLST, multi locus sequence type; WGS, whole genome sequencing; AMP, ampicillin; AUG, amoxicillin-clavulanic acid; AZI, azithromycin; C, chloramphenicol; CIP, ciprofloxacin; CRO, ceftriaxone; TZ, ceftazidime; IMI, imipenem; NAL, nalidixic acid; SM, streptomycin; TE, tetracycline; T/S, trimethoprim-sulfamethoxazole; S3, sulfonamides. *Intermediate susceptibility to antimicrobial agent. **Not identified (NI).

The occurrence of genes conferring resistance to β-lactams was also investigated among the genomes of 17 ESBL-negative isolates. According to CARD, all of them displayed the similar two amino acid substitutions in PBP3 (S357N and D350N), which were previously detected in all ESBL-positive isolates (Tables 2, 3). These two substitutions were the only detectable β-lactam resistance mechanisms among the majority of ESBL-negative isolates (9/17, 52.94%). Surprisingly, however, four out of the nine above isolates were susceptible to all β-lactams tested. The other four – showed intermediate or full resistance to ampicillin. And the remaining *S*. Typhimurium A_1153 isolate displayed resistance to ampicillin and amoxicillin-clavulanic acid, intermediate resistance to imipenem, and full resistance to azithromycin (MIC 32 μg/mL). Among the remaining eight ESBL-negative isolates with the two amino acid substitutions in PBP3, additional β-lactam resistance genes were detectable (Table 3). Their prevalence was as follows: the *bla*_OXA–__1_ gene was identified in three isolates (17.65%, 3/17), the *bla*_TEM–__1_ gene in the other three isolates (17.65%, 3/17), and the *bla*_CTX–M–__3_ and *bla*_CTX–M–__15_ genes were encountered as singletons (5.88% each). All these eight isolates were at least resistant to ampicillin. All isolates harboring *bla*_OXA–__1_ were *S*. Typhimurium, expressing resistance to amoxicillin-clavulanic acid. One of these isolates, *S*. Typhimurium A_3889, displayed additional resistance to ceftriaxone and ceftazidime. From three isolates carrying *bla*_TEM–__1_, two showed intermediate resistance to amoxicillin-clavulanic acid (*S*. Kentucky A_478 and *S*. Typhimurium G_1150), while one isolate (*S*. Enteritidis A_588) was susceptible to this antibiotic. *S*. Enteritidis A_7201 harboring the *bla*_CTX–M–__3_ gene showed intermediate resistance to ceftriaxone, while *S*. Enteritidis G_104 with *bla*_CTX–M–__15_ was resistant to ceftriaxone and ceftazidime and displayed intermediate resistance to amoxicillin-clavulanic acid.

**TABLE 3:** Profiles of genes conferring resistance to β-lactam antibiotics identified *in silico* in 17 ESBL-negative MDR isolates of NTS.

Profile no.	Number of isolates (%)	Isolate ID	Serotype	Year of isolation	MLST	Antimicrobial resistance phenotype	Antimicrobial resistance genes identified by WGS		
							Resistance to β-lactams	Resistance to quinolones/fluoroquinolones	Resistance to other classes of antimicrobials
1	9 (52.94%)	A_1153	*S*. Typhimurium	2016	328	AMP, AUG, AZI, IMI*	*ftsI* (S357N, D350N)	*gyrA (*D87N*)*	*aac(6*′*)-Iaa*
		A_4649	*S*. Derby	2016	40	CIP, NAL, SM, S3, TE	*ftsI* (S357N, D350N)	*qnrB10*	*aac(6*′*)-Iy*, *aadA2*, *sul1*, *tet(A)*
		A_4970	*S*. Derby	2016	40	CIP, NAL, SM, S3, TE	*ftsI* (S357N, D350N)	*qnrB10*	*aac(6*′*)-Iy*, *aadA2*, *sul1*, *tet(A)*
		A_5064	*S*. Typhimurium	2016	19	SM, S3, TE	*ftsI* (S357N, D350N)	NI**	*aac(6*′*)-Iaa*, *aph(3*′′*)-Ib*, *aph(6)-Id*, *sul2*, *tet(A)*
		A_6059	*S*. Enteritidis	2016	11	AMP, NAL, C	*ftsI* (S357N, D350N)	*gyrA (*D87N*)*	*aac(6*′*)-Iaa*, *catII*, *tet(D)*
		A_6187	*S*. Enteritidis	2016	11	AMP*, C, TE	*ftsI* (S357N, D350N)	NI	*aac(6*′*)-Iaa*, *catI*, *tet(A)*
		A_7187	*S*. Newport	2013	31	AMP*, NAL, SM*, S3, T/S, TE	*ftsI* (S357N, D350N)	*aac(6*′*)-Ib-cr*	*aac(6*′*)-Iy*, *aadA16*, *arr3*, *dfrA27*, *tet(A)*, *sul1*
		A_8239	*S*. Typhimurium	2016	19	SM, S3, TE	*ftsI* (S357N, D350N)	NI	*aac(6*′*)-Iaa*, *aph(3*′′*)-Ib*, *aph(6)-Id*, *sul2*, *tet(A)*
		G_311	*S*. Typhimurium	2016	36	AMP, AUG*, NAL, C, S3	*ftsI* (S357N, D350N)	NI	*aac(6*′*)-Iaa*, *aph(3*′′*)-Ib*, *floR*, *sul2*
2	3 (17.65%)	A_1722	*S*. Typhimurium	2012	328	AMP, AUG, CIP*, NAL, C, SM*, TE, S3, T/S	*bla*_OXA–__1_, *ftsI* (S357N, D350N)	*gyrA (*D87N*)*	*aac(6*′*)-Iaa*, *aadA*, *aph(3*′*)-Ia*, *catI*, *sul1*, *tet(B)*, *tetR*
		A_3889	*S*. Typhimurium	2012	328	AMP, AUG, CRO, TZ*, CIP*, NAL, C, SM*, S3, TE	*bla*_OXA–__1_, *ftsI* (S357N, D350N)	*gyrA (*D87N*)*	*aac(6*′*)-Iaa*, *aadA*, *aph(3*′*)-Ia*, *catI*, *sul1*, *tet(B)*, *tetR*
		A_5923	*S*. Typhimurium	2011	328	AMP, AUG, AZI, CIP*, NAL, C, SM, S3, T/S	*bla*_OXA–__1_, *ftsI* (S357N, D350N)	*gyrA (*D87N*)*	*aac(6*′*)-Iaa*, *aadA*, *aph(3*′*)-Ia*, *dfrA19*, *sul1*
3	3 (17.65%)	A_478	*S*. Kentucky	2016	198	AMP, AUG*, CIP*, NAL	*bla_TEM–__1_*, *ftsI* (S357N, D350N)	*gyrA (*S83F*)*, *parC* (S80I)	*aac(6*′*)-Iy*
		A_588	*S*. Enteritidis	2016	11	AMP, CIP*, NAL, SM*, S3, T/S	*bla_TEM–__1_*, *ftsI* (S357N, D350N)	*gyrA (*D87N*)*	*aac(6*′*)-Iy*, *aadA5*, *dfrA17*
		G_1150	*S*. Typhimurium	2016	19	AMP, AUG*, SM, S3, T/S	*bla_TEM–__1_*, *ftsI* (S357N, D350N)	NI	*aac(6*′*)-Iaa*, *aph(3*′*)-Ia*, *aph(3*′′*)-Ib*, *aph(6)-Id*, *dfrA5*, *sul2*
4	1 (5.88%)	A_7201	*S*. Enteritidis	2016	11	AMP, CRO*, CIP, NAL	*bla*_CTX–M–__3_, *ftsI* (S357N, D350N)	NI	*aac(6*′*)-Iaa*, *rpoB*
5	1 (5.88%)	G_104	*S*. Enteritidis	2016	11	AMP, AUG*, CRO, TZ, S3	*bla*_CTX–M–__15_, *ftsI* (S357N, D350N)	*qnrS1*	*aac(6*′*)-Iaa*

ESBL, extended-spectrum beta-lactamase; MDR, multidrug-resistant; NTS, non-typhoidal Salmonella; MLST, multi locus sequence type; WGS, whole genome sequencing; AMP, ampicillin; AUG, amoxicillin-clavulanic acid; AZI, azithromycin; C, chloramphenicol; CIP, ciprofloxacin; CRO, ceftriaxone; TZ, ceftazidime; IMI, imipenem; NAL, nalidixic acid; SM, streptomycin; TE, tetracycline; T/S, trimethoprim-sulfamethoxazole; S3, sulfonamides. *Intermediate susceptibility to antimicrobial agent. **Not identified (NI).

Interestingly, in two *S*. Typhimurium isolates with a similar AMR profile toward eight AM classes, the resistance gene repertoire was also identical but with one exception. The *bla*_CTX–M–__5_ gene was detected in an ESBL-producing strain, A_5962, while in an ESBL-negative A_3889 strain this gene could not be detected.

Inspection of the genomic sequences with the use of CARD revealed an interesting situation with the uniform presence of certain putative AMR genes in the genomes of all NTS strains sequenced (Supplementary Table 4). The most notable case included the presence of ampC-type β-lactamases (CARD accession numbers ARO:3004612 and ARO:3004611) in the genomes of all NTS isolates, irrespectively of the β-lactam resistance phenotype. Presumably, these are the silent β-lactamase genes since they are present in the strains sensitive to all β-lactams tested.

### Resistance to Quinolones and Fluoroquinolones

We detected a single mutation in the *gyrA* gene (D87N), which is associated with resistance to quinolones, in all ESBL-producing isolates. This is consistent with the nalidixic acid-resistant phenotype of these isolates. The mutated *gyrA* (D87N) gene, identified in 35.29% of ESBL-negative isolates (6/17), was the most prevalent gene associated with resistance to quinolones and fluoroquinolones. The lower prevalence of this mutation in the ESBL-negative compared to ESBL-positive isolates (35.29% vs. 100%; *p* < 0.0001) is consistent with a higher susceptibility of the former to nalidixic acid compared to the latter [35.29% (6/17) vs. 0% (0/19); *p* < 0.01]. The mutated *gyrA* (D87N) allele was detected in four from five ESBL-negative isolates with intermediate resistance to ciprofloxacin. In one isolate, *S*. Kentucky A_478, *gyrA* (S83F) allele was combined with another ciprofloxacin-resistance mutation, in the *parC* (S80I) gene. In two isolates, *S*. Enteritidis A_6059 and *S*. Newport A_7187, which were resistant to nalidixic acid but susceptible to ciprofloxacin, we identified the *gyrA* (D87N) allele and the *aac(6′)-Ib-cr* gene, respectively. Besides, the *qnrB10* gene was identified in two *S*. Derby isolates that displayed resistance to ciprofloxacin. Remarkably, no any known quinolone/fluoroquinolone resistance determinant in the CARD database could be detected in *S*. Enteritidis A_7201 isolate, which was resistant to ciprofloxacin and nalidixic acid, as well as in *S*. Typhimurium G_311 isolate, which was resistant to nalidixic acid but susceptible to ciprofloxacin. Thus, resistance to quinolones/fluoroquinolones among the ESBL-negative isolates was mainly associated with the *gyrA* (D87N) allele in *S*. Typhimurium and *S*. Enteritidis serovars, whereas in other serotypes it was associated with different genetic background such as the presence of *qnrB10* in *S*. Derby and *aac(6′)-Ib-cr* in *S*. Newport isolates or the combination of *parC* (S80I) and *gyrA* (S83F) alleles in the *S*. Kentucky isolate.

### Other AMR Genes

We then inspected genomes of 19 ESBL-producers for genes conferring resistance to AMs other than β-lactams and quinolones/fluoroquinolones (Table 2). Resistance to trimethoprim-sulfamethoxazole, which was identified in 26.32% (5/19) of these isolates, was associated with the presence of *dfrA5* in two isolates (A_6004 and A_8011), combination of *dfrA19* and *sul1* – in two isolates (A_3040 and A_3246), and *dfrA14* – in one isolate (A_126). In four tetracycline-resistant isolates (A_3040, A_3246, A_3725, and A_5962) among 19 ESBL-producers (21.05%), the presence of *tet*(B) and *tetR*(B) was detected. These four isolates were also resistant to chloramphenicol and streptomycin. Genetic background for these resistance included the *catI* gene, which confers resistance to chloramphenicol, and the combination of *aac(6′)-Iaa, aadA*, and *aph(3′)-Ia*, which confer resistance to aminoglycosides. In one of these isolates, A_3246, which was the only gentamicin-resistant isolate in this study, additional resistance genes were identified. These were: the *ant(2′′)-Ia* gene, which is associated with resistance to gentamicin, tobramycin, and kanamycin (Cox et al., 2015) and the *catB3* gene, which confers resistance to chloramphenicol. Interestingly, the *aac(6′)-Iaa* gene was also identified in aminoglycoside-susceptible ESBL-producing isolates (13/19, 68.42%). It was a singleton in these aminoglycoside-susceptible cases though, not the combination of aminoglycoside-resistance conferring genes as described above for the aminoglycoside-resistant isolates.

Genomes of ESBL-negative isolates were also inspected for genes conferring resistance to other AM classes (Table 3). Among the ESBL-negative isolates, we detected a higher frequency of genes associated with resistance to folate pathway inhibitors (64.71%, 11/17) (Table 3) compared to ESBL-producers (36.84%, 7/19) (Table 2). One of these genes, *sul2*, was detected in ESBL-negative isolates only (*p* < 0.05). These findings are consistent with a higher level of sulphonamide-resistance phenotype in ESBL-negative isolates (12/17, 70.59%) compared to ESBL-producers (7/19, 36.84%). In ESBL-negative isolates, the most prevalent was the *sul1* gene, detected in six isolates (6/17, 35.29%), followed by *sul2* gene, identified in four isolates (4/17, 23.53%). All these isolates were resistant to at least sulphonamide. The following gene combinations were identified in three out of five ESBL-negative strains, resistant to trimethoprim-sulfamethoxazole: *dfrA19* and *sul1* (in *S*. Typhimurium A_5923), *dfrA27* and *sul1* (in *S*. Newport A_7187), *dfrA5* and *sul2* (in *S*. Typhimurium G_1150). The *dfrA17* and *sul1* genes were detected as singletons in the remaining two isolates resistant to trimethoprim-sulfamethoxazole (*S*. Enteritidis A_588 and *S*. Typhimurium A_1722, respectively). Notably, no known corresponding resistance genes could be detected in a sulphonamide-resistant *S*. Enteritidis G_104.

Aminoglycoside resistance genes were identified in the genomes of all ESBL-negative isolates sequenced (Table 3). The *aac(6′)-Iaa* gene was the most prevalent in ESBL-negative isolates (12/17, 70.59%) and six of them were susceptible to streptomycin. In six streptomycin-resistant ESBL-negative isolates, we identified several combinations of genes that may confer resistance to aminoglycosides. These were: (i) combination of *aac(6′)-Iaa*, *aph(3′′)-Ib*, and *aph(6)-Id* in two *S*. Typhimurium isolates, A_5064 and A_8239; (ii) combination of *aac(6′)-Iy* and *aadA2* in two *S*. Derby isolates, A_4649 and A_4970; (iii) combination of *aac(6′)-Iaa*, *aph(3′)-Ia*, *aph(3′′)-Ib*, and *aph(6)-Id* in a *S*. Typhimurium G_1150 strain; and (iv) combination of *aac(6′)-Iaa*, *aadA*, and *aph(3′)-Ia* in a *S*. Typhimurium A_5923 strain. The latter gene combination was also detected in two *S*. Typhimurium isolates, A_3889 and A_1722, which displayed intermediate resistance to streptomycin. Besides, the following combinations of aminoglycoside resistance genes were identified in two other isolates with intermediate resistance to streptomycin: *aac(6′)-Iy* and *aadA5* in *S*. Enteritidis A_588 and *aac(6′)-Iy*, *aac(6′)-Ib-cr* and *aadA16* in *S*. Newport A_7187. Notably, the combination of *aac(6′)-Iaa* and *aph(3′′)-Ib* was detected in a streptomycin*-*susceptible *S*. Typhimurium G_311 strain. The *aph(3′′)-Ib* gene was found to be more prevalent in ESBL-negative isolates compared to ESBL-producers (23.53% vs. 0%; *p* < 0.05).

The most prevalent tetracycline resistance gene among the ESBL-negative isolates was *tet*(A) (6/17, 35.29%), which was detected in six out of eight tetracycline-resistant strains (Table 3). The gene was carried by two *S*. Typhimurium strains (A_5064 and A_8239), two *S*. Derby strains (A_4649 and A_4970), *S*. Enteritidis A_6187 strain, and *S*. Newport A_7187 strain. Notably, the prevalence of the *tet*(A*)* gene was higher in ESBL-negative isolates compared to ESBL-producers (35.29% vs. 0%; *p* < 0.05). Besides, the *tet*(B) and *tetR*(B) gene pairs were found in the other two tetracycline*-*resistant *S*. Typhimurium strains A_1722 and A_3889. Among the six chloramphenicol-resistant ESBL-negative isolates, three harbored *catI* (*S*. Typhimurium A_1722, *S*. Typhimurium A_3889, and *S*. Enteritidis A_6187), one – the *catII* gene (*S*. Enteritidis A_6059), and one – the *floR* gene (*S*. Typhimurium G_311). No known phenicol resistance determinant, however, could be found in the chloramphenicol-resistant *S*. Typhimurium A_5923.

As for other AMR genes detected in the genomes (Supplementary Table 4), the corresponding AMR phenotypes were not tested. The literature available regarding, for example, fosfomycin resistance genes, suggest their much lower occurrence in *S. enterica* (McMillan et al., 2019). Resistance to polymyxins among *S. enterica* also appears to be not too frequent (Lima et al., 2019). Thus, genotypic detection of AMR genes may not necessarily translate into the corresponding phenotype.

### Mobile Genetic Elements Associated With ESBL Production

PlasmidFinder 2.1 tool (Carattoli et al., 2014) was employed to predict plasmids in the genomes of 19 ESBL-producing isolates. Plasmid-specific sequences were detected in 17 isolates, while no plasmid sequences were predicted in two ESBL-producer isolates. The latter isolates were collected in 1996 (A_60 and A_684). PlasmidFinder 2.1 tool predicted two plasmid sequences in the genomes of 16 ESBL-producer isolates. They were located within the same contigs where the *bla*_CTX–M–__5_ gene was also located. The two plasmid replicons in our genomic sequences were pHAD28 (GenBank accession number KU674895) and Col440II/ColRNAI-like, which were predicted together with 93.89% and 85% DNA similarity, respectively. In the genome of one ESBL-producer isolate (A_3017), these two plasmid replicons were also predicted together in one contig, but the *bla*_CTX–M–__5_ gene was in a different contig.

All 16 contigs, which contained the two plasmid replicons predicted by PlasmidFinder 2.1 tool and the *bla*_CTX–M–__5_ gene, were analyzed by BLAST^9^. The CTX-M-5-encoding plasmids were detected in all these contigs. In particular, pCTXM5-1358 (JX017308) was detected in 13 contigs, and pCTXM5-637 (JX017306) was detected in three contigs. Thus, plasmid-encoded CTX-M-5 production was detectable in 16 ESBL-producers. These observations suggest that 16 out of 19 ESBL-producing isolates in our study carried the CTX-M-5- encoding plasmids, which may contribute to the dissemination of ESBL among NTS in the region. In general, the size of 14 out of 16 contigs was well within the range of pCTXM5 plasmids, from 7,700 to 7,699 bp. Two contigs, however, had much larger sizes, 41,849 and 982,461 bp. These could be assembly artifacts or genuine integration of pCTXM5 plasmids into a larger plasmid (41,849 bp) or the chromosome (982,461 bp).

In one ESBL-producer isolate, A_3017, one contig contained the above mentioned two plasmid replicons, pHAD28 and Col440II/ColRNAI-like, but not the *bla*_CTX–M–__5_ gene. Nevertheless, the contig produced a partial overlap with the pCTXM5-1358 plasmid. In two ESBl-producers, however, no plasmid replicons could be predicted by the PlasmidFinder 2.1 tool. According to our unpublished data, in these two isolates, plasmids cannot be detected on agarose gels as well. However, analysis of contigs containing the *bla*_CTX–M–__5_ gene using MobileElementFinder (v1.02) tool (Johansson et al., 2020) revealed the insertion sequence ISEc9 (AJ242809) (IS1380 family) at a distance of 19 bp from the *bla*_CTX–M–__5_ gene similar to the reported in CTX-M-5-encoding plasmids (Kozyreva et al., 2014).

Genomes of all ESBL-producing isolates were interrogated for the presence of genomic islands using GIPSy (Soares et al., 2016) and IslandViewer4 (Bertelli et al., 2017) tools, as well as by screening for the presence of SGI1 in genomic sequences as described before (Siebor and Neuwirth, 2013, 2014). No genomic islands could be detected in ESBL-producing isolates from Armenia using any of the approaches.

### Mobile Genetic Elements Associated With Other AMR

In contigs of ESBL producers, which carried the *bla*_CTX–M–__5_ gene, only the CTX-M-5-encoding plasmid replicons were detected but no other plasmid replicons. However, in other contigs of five ESBL producers some other plasmid replicons were predicted, and these replicons were associated with other AMR genes (Supplementary Table 5). In three isolates (A_3040, A_3246, and A_5962) the IncFII(pRSB107) (AJ851089), IncFIB (AP001918), and IncFIA (AP001918) replicons were detectable simultaneously in the same contigs. All these contigs also carried the *cat1*, *tet*(B) and *tetR* genes. In the latter two isolates the following additional AMR genes were located within the same contig: *aadA*, *aph(3′)-Ia*, *bla*_OXA–__1_, and *sul1*. In should be emphasized here, that these three ESBL producers were resistant to 8 classes of AMs. The well-known penta-resistant phenotype (resistance to ampicillin, chloramphenicol, streptomycin/spectinomycin, sulphonamides, and tetracycline) occurs in *S*. Typhimurium DT104 strains harboring SGI1 (Boyd et al., 2001). However, DT104 as well as SGI1 markers were not detected in our ESBL-producing isolates. In the other two ESBL producers, A_6004 and A_8011, IncN (AY046276) plasmids were predicted. These plasmid replicons resided within the same contigs where the *bla*_CTX–M–__15_ and *dfrA5* genes were located.

In the genomes of two ESBL-negative isolates, A_1722 and A_5923, the plasmid replicons described above [IncFII(pRSB107) (AJ851089), IncFIB (AP001918), and IncFIA (AP001918)] were also detectable. They resided within the contigs where other AMR genes were located. These two ESBL-negative *S*. Typhimurium isolates showed resistance to up to 7 classes of AMs, including a penta-resistant phenotype typical of *S*. Typhimurium DT104. However, markers for the phage type DT104 were absent in these two isolates. Among other ESBL-negative isolates, in two *S*. Derby serovars, Col(pHAD28) (KU674895) plasmid replicons were predicted. The replicons were located within the same contigs that also carried the *qnrB10* genes. This plasmid replicon was also detected in *S*. Typhimurium isolate A_5064, where it resided on the same contig with the *aph(3′′)-Ib*, *aph(6)-Id*, *sul2*, and *tet*(A) genes. This AMR gene profile was also detected in another ESBL-negative *S*. Typhimurium isolate, A_8239, but in this case it was associated with the IncQ1 (M28829) plasmid replicon within the same contig. Besides, in one *S*. Enteritidis isolate (A_588) the IncX1 (EU370913) plasmid replicon was predicted, which was genetically linked with the *aadA5*, *bla*_TEM–__1_ and *dfrA17* genes on the same contig. The *bla*_TEM–__1_ gene was located within the Tn*2* (HM749967.1) unit transposone. In addition, in *S*. Newport serovar, the IncN (AY046276) plasmid replicon was predicted, which resided within the same contig where the following six AMR genes were detected: *aac(6′)-Ib-cr*, *aadA16*, *arr3*, *dfrA27*, *sul1*, and *tet*(A).

Among the ESBL-negative isolates, the genome of *S*. Kentucky A_478 was positive for SGI1. The contig of this isolate, which contains the genomic island, was analyzed by BLAST and a structure similar to SGI1-P type of SGI (Doublet et al., 2008) that contains an IS26 flanked composite transposon carrying the *bla*_TEM–__1_ gene was detected. In all other ESBL-negative isolates in this study SGIs were not detectable. The only phage type DT104 isolate from Georgia, S. Typhimurium G_311, also had no detectable SGI1. This isolate exhibited resistance to ampicillin, chloramphenicol, and sulphonamides but was susceptible to streptomycin and tetracycline.

Thus, ESBL-producer phenotype in NTS isolates in our study is associated with plasmid-encoded CTX-M-5 production. A diverse range of other plasmid replicons are associated with other AMRs, including the multidrug resistance phenotype conferring resistance to three and more AM classes.

## Discussion

In this work, we investigated the prevalence of AMR among 291 clinical NTS strains recovered from patients in Armenia and Georgia. The prevalence of MDR among these isolates was 19.93% and 15% in each respective country. Although the MDR phenotype could be found in all five NTS serotypes investigated, the most prevalent MDR serotype was *S*. Typhimurium, accounting for 84.21% of all isolates. According to the genomic-based sequence typing, ST328 was the most prevalent sequence type among the MDR *S*. Typhimurium strains isolated in Armenia (22/25, 88%). Interestingly, this ST was not detected among the NTS isolates from Georgia. Other MDR *S*. Typhimurium isolates were typed as ST19 (three isolates) and ST36 (one isolate). We also noted a significant correlation between the AMR profile and ST/serotype in MDR NTS isolates. Among these isolates, about third of them were resistant to five or more AM classes (19/57, 33.33%), which were almost exclusively represented by *S*. Typhimurium ST328 strains, except for one *S*. Newport strain belonging to ST31. Worryingly, five *S*. Typhimurium ST328 strains were resistant to eight AM classes thus severely limiting treatment options.

This study is the first description of the MDR ESBL-producing NTS isolates of human origin in the region. All of them are from Armenia, whereas the corresponding three MDR NTS isolates from Georgia were all ESBL-negative, may be due to the small number of isolates from Georgia studied in this work. All ESBL-producers were identified as *S.* Typhimurium and were recovered between 1996 and 2014. From the 19 ESBL-producing *S*. Typhimurium isolates sequenced, 18 were assigned to ST328, while the ST of one isolate could not be clearly determined. It should be noted that no ESBL-production was detectable among the *S*. Typhimurium isolates collected in 2016 and only one of these isolates, displaying MDR phenotype but ESBL-negative, was assigned to ST328. In general, however, the majority of clinical MDR *S*. Typhimurium isolates from Armenia were ESBL-producers (37/46, 80.43%). Moreover, the ESBL-producer phenotype was present in four out of five *S*. Typhimurium ST328 isolates displaying resistance to eight classes of AMs. Expectedly, ESBL-producers demonstrated a high-level resistance toward β-lactams, while the ESBL-negative isolates were significantly more sensitive to these antibiotics.

Determination of the genetic background for AMR phenotypes in the 36 MDR isolates was based on *in silico* prediction of AM resistance determinants in the genomic sequences generated with WGS. Initially we identified the genes of resistance to β-lactam antibiotics in 19 ESBL-producers compared to 17 ESBL-negative isolates. The same amino acid substitutions S357N and D350N in PBP3 encoded by the *ftsI* gene (Misawa et al., 2018), were found in all MDR NTS isolates, regardless of the ESBL-production phenotype. Interestingly, four strains with these substitutions were sensitive to all β-lactams tested. This poses a question whether these substitutions in PBP3 are actually involved in a full β-lactam resistance phenotype in NTSs or these are natural SNPs with very low phenotypic expression. The *bla*_CTX–M–__5_ gene, on the contrary, was firmly and unequivocally associated with ESBL resistance, being detected in all ESBL-producing isolates but not in the ESBL-negative isolates. All isolates harboring other β-lactamase genes (*bla*_CTX–M–__3_, *bla*_CTX–M–__15_, *bla*_OXA–__1_, and *bla*_TEM–__1_) and the *ftsI* allele with S357N and D350N substitutions, but without the concurrent presence of the *bla*_CTX–M–__5_ gene, displayed an ESBL-negative phenotype (Table 3). This suggests the key role played by the *bla*_CTX–M–__5_ gene in the ESBL-producing phenotype of NTS. These findings are also consistent with the results of PCR-testing of 21 MDR isolates that were not subjected to WGS. In all these isolates with the ESBL-producer phenotype, we detected CTX-M β-lactamases, whereas other β-lactamases were not detected.

The *bla*_CTX–M–__5_ gene has been initially identified in ESBL-producing *S*. Typhimurium isolates in 1998 (Bradford et al., 1998). The gene encodes CTX-M-5, a β-lactamase, which preferentially hydrolyses cefotaxime over ceftazidime and which is efficiently inhibited by clavulanic acid, tazobactam, and sulbactam (Bradford et al., 1998; Tzouvelekis et al., 2000). The *bla*_CTX–M–__5_ gene is predominantly located on plasmids, small non-self-transferable or conjugative (Bradford et al., 1998; Edelstein et al., 2004; Kozyreva et al., 2014). Since the first publication in 1998, infections caused by *S*. Typhimurium ST328 carrying *bla*_CTX–M–__5_ gene have been reported in many countries of the world (Gazouli et al., 1998; Tassios et al., 1999; Edelstein et al., 2004; Tapalski et al., 2007; Sjölund-Karlsson et al., 2008, 2011; Krauland et al., 2009). Our findings confirm the presence of *bla*_CTX–M–__5_-positive *S.* Typhimurium ST328 in samples from salmonellosis patients in Armenia in 1996-2014. Moreover, *bla*_CTX–M–__5_-positive *S.* Typhimurium ST328 is generally associated with the carriage of pCTXM5-1358 and pCTXM5-637 plasmids. These mobilizable plasmids have been previously found as being associated with the clonal distribution of ST328 in several FSU countries from 1996 to 2009 (Kozyreva et al., 2014). These isolates exhibited a hypermutable phenotype, which may contribute to quinolone/fluoroquinolone resistance mutations.

Fluoroquinolone susceptibility in MDR NTS isolates is almost equal among the ESBL-producing and ESBL-negative strains, with the corresponding frequencies of 56.76 and 55.00%. As for the older representative of this class, nalidixic acid, however, all ESBL-producers and 70% of ESBL-negative isolates were resistant to it. All the MDR ESBL-producers sequenced were identified as *S*. Typhimurium and in all of them this resistance phenotype was exclusively associated with a single mutation in the *gyrA* gene (D87N). In ESBL-negative MDR NTS isolates, however, a broader range of the corresponding resistance mechanisms as well as taxonomic/ST affiliations were revealed. In particular, *gyrA* (D87N) in *S*. Typhimurium and *S*. Enteritidis isolates, *qnrB10* in *S*. Derby isolates, *gyrA* (S83F) and *parC* in *S*. Kentucky, and *aac(6′)-Ib-cr* in *S*. Newport.

During analysis of WGS data for the presence of AMR genes we encountered a number of cases, where AMR genes can be detected in strains that are apparently susceptible to the corresponding AMs. These are the cases, for example, with amino acid substitutions in PBP3 found in β-lactam susceptible strains, the presence of the *aac(6′)-Iaa* gene in aminoglycoside-susceptible strains, or detection of ampC-type β-lactamases in all genomes sequenced. This suggest that certain AMR genes are not expressing the corresponding phenotype and remain silent. This pool, however, could be potentially activated under selective pressure of AMs, by incorporation of the silent structural genes under the control of external promoters, for example, of integrons.

Our findings indicate that a substantial proportion of human NTS isolates in the region are multidrug-resistant. In many cases genes conferring the MDR phenotype are associated with mobile genetic elements, in particular plasmids. The plasmids detected in this work belong to a variety of incompatibility groups and thus can coexist in the same host allowing to carry a broader arsenal of AMR genes. Among resistances, especially worrying is a high-level of resistance toward the third generation cephalosporins and fluoroquinolones, which are the first-line drugs for the empiric therapy of acute salmonellosis. In addition, the non-susceptibility to the alternative treatment options such as azithromycin and trimethoprim-sulfamethoxazole was also found to be substantial, 14.04 and 17.54% correspondingly, among the MDR isolates. Among the AMs tested in this work, only imipenem demonstrated activity against all ESBL-producing isolates and all isolates with resistance to eight classes of AMs. Imipenem, therefore, remains one of the last-resort AMs for treatment of severe *Salmonella* infections. Detection of non-susceptibility to this antibiotic in a *S*. Typhimurium ST328 strain, which was isolated in 2016 and which is ESBL-negative, however, poses a question how long its efficiency could be maintained. Continuous surveillance of resistance to carbapenems is necessary to preserve their efficacy in treatment of NTS and other infections (Fernández et al., 2018).

The results indicated the limitations of current AM therapy to control infections caused by MDR isolates of NTS, especially belonging to serotype Typhimurium and ESBL-producing. Inadequate initial empirical treatment is an important risk factor associated with worse clinical outcomes in patients. Early diagnostic, serotype identification and AM susceptibility testing are crucial to avoid further complications of salmonellosis. The results of this work once again emphasized the importance of more stringent measures to identify possible sources of NTS infections, especially caused by MDR and ESBL-producing isolates, in order to prevent the spread of epidemically successful clones of NTS displaying resistance to multiple antimicrobial agents.

The results were partly presented at the IACMAC XX International Congress “Antimicrobial Therapy” Moscow, May 23–25, 2018.

## Data Availability Statement

The datasets generated for this study can be found in online repositories. The names of the repository/repositories and accession number(s) can be found below: https://www.ebi.ac.uk/ena, Projects PRJEB36290, PRJNA656406, PRJNA656411, and PRJNA656415.

## Author Contributions

AS, ZK, and RA designed the study. AS, KA, MZ, ZG, AM, AH, EK, and KM performed the experiments. AS, MZ, and RA analyzed the data and wrote the paper. AA, NC, J-PP, and RA provided critical review and editing of the manuscript. All authors contributed to the article and approved the submitted version.

## Conflict of Interest

The authors declare that the research was conducted in the absence of any commercial or financial relationships that could be construed as a potential conflict of interest.

## Footnotes

1 http://www.microbesng.uk

2 www.denglab.info/SeqSero

3 https://card.mcmaster.ca/analyze/rgi

4 https://cge.cbs.dtu.dk/services/PlasmidFinder/

5 https://cge.cbs.dtu.dk/services/MobileElementFinder/

6 http://www.pathogenomics.sfu.ca/islandviewer/

7 http://blast.ncbi.nlm.nih.gov/Blast.cgi

8 http://www.graphpad.com/quickcalcs/contingency1.cfm

9 http://blast.ncbi.nlm.nih.gov/Blast.cgi
